# Improving patient access to educational resources: the development of an educational resource for congenital adrenal hyperplasia

**DOI:** 10.1186/1687-9856-2013-S1-P132

**Published:** 2013-10-03

**Authors:** Irene Mitchelhill, Jennie King, Nat Jackson, Pamela Dawes

**Affiliations:** 1 Sydney Children's Hospital, Sydney, Australia; 2 University of Sydney Nursing School, NSCCH, Sydney, Australia; 3 Jacksonspeed Media Productions, Sydney, Australia

## 

CAH, a chronic condition with essentially life threatening aspects, is a frightening concept for families to come to terms with, even more so in the isolation of country areas. Having adequate knowledge and understanding is important in maintaining a sense of normality for these families and is an essential component to management both medically and within the family.

The ultimate goal of this study was to improve patient access to education and support, and improve patient outcomes though increased knowledge and understanding. The principle aim of this study was to extend the education service we provide for our patients locally at specialist centres, to the patients who live in isolated regions of NSW and Australia wide. A comprehensive psychosocial education program (PEP) which was developed for patients and families with Congenital Adrenal Hyperplasia (CAH) and run at our local centre, was developed into an audiovisual format. In doing so, the program could be facilitated by one experienced health professional, therefore eliminating the need for a team of specialists to travel country areas, which is logistically difficult and considerably costly.

Outreach services in NSW Australia, often provide only specialist medical care, with country families having only limited access to education, counselling and support services, that is routinely provided in metropolitan centres. In order to meet their needs the development of this innovative education program into an audiovisual format was planned and developed in order to provide an educational resource not only for patients and families, but also for clinicians.

The PEP has been run 4 times with the program designed to cover the essential information required for families to know and understand the condition. Four 20 minutes sessions: What is CAH, Adolescent/Adult Issues, Psychosocial Issues and Sick Day Management, are followed by a 4 minute session on how to inject Hydrocortisone.

The process of developing an audiovisual educational tool will be discussed and the essential factors which need to be considered, in developing such programs into an audiovisual format will be discussed in detail.

This program is used in conjunction with the CAH Knowledge Questionnaire[1] (CAHKAQ) developed to evaluate our education process.
